# Supplementary material for: Enhanced Efficacy of Gastric Cancer Treatment through Targeted Exosome Delivery of 17-DMAG Anticancer Agent
Source: Int J Mol Sci. 2024 Aug 12;25(16):8762. doi: 10.3390/ijms25168762 (PMC11354984; doi:10.3390/ijms25168762)
Supplement: Supplementary file 1 [file ijms-25-08762-s001.zip › ijms-3037390-supplementary.pdf]

Supplement data. Weight change of xenograft mouse after treatment

Average weight through days after treatment

AGS	0	5	10	15	20	25	30
Ct	25.17	25.33	24.73	25.80	25.53	25.20	25.87
Ex	25.60	25.23	25.70	25.30	25.37	25.20	25.33
Ex(D)	24.67	25.50	26.23	24.93	26.00	24.37	25.30
tEx	25.63	25.97	26.73	26.23	26.27	26.43	25.97
tEx(D)	25.30	25.03	25.90	26.50	25.70	25.63	25.13

Standard deviation

AGS	0	5	10	15	20	25	30
Ct	0.60	0.42	0.21	0.53	0.84	0.60	0.76
Ex	0.35	0.55	0.44	0.62	0.60	0.75	0.61
Ex(D)	0.45	0.15	0.64	0.68	0.64	0.51	0.55
tEx	0.58	0.62	0.45	0.70	0.70	1.01	1.04
tEx(D)	0.62	0.15	1.01	0.50	0.36	0.47	0.51

Average weight through days after treatment

MKN45	0	5	10	15	20	25	30
Ct	23.37	24.03	22.60	21.77	21.60	20.07	20.53
Ex	23.43	23.33	22.87	22.77	22.20	21.40	21.63
Ex(D)	23.13	23.17	22.53	22.60	21.50	20.40	20.10
tEx	23.23	23.10	22.80	22.77	22.27	21.23	21.67
tEx(D)	24.23	24.20	23.43	23.53	23.30	22.43	22.83

Standard deviation

MKN45	0	5	10	15	20	25	30
Ct	1.46	2.32	3.03	2.90	2.21	2.18	2.08
Ex	0.21	0.49	1.20	1.21	0.75	0.36	0.85
Ex(D)	1.29	1.31	1.12	1.14	1.13	0.98	0.89
tEx	2.81	3.22	2.85	2.85	3.25	3.03	2.20
tEx(D)	3.33	3.00	2.70	1.80	2.76	2.00	1.67

Supplement data. Weight change of xenograft mouse after treatment

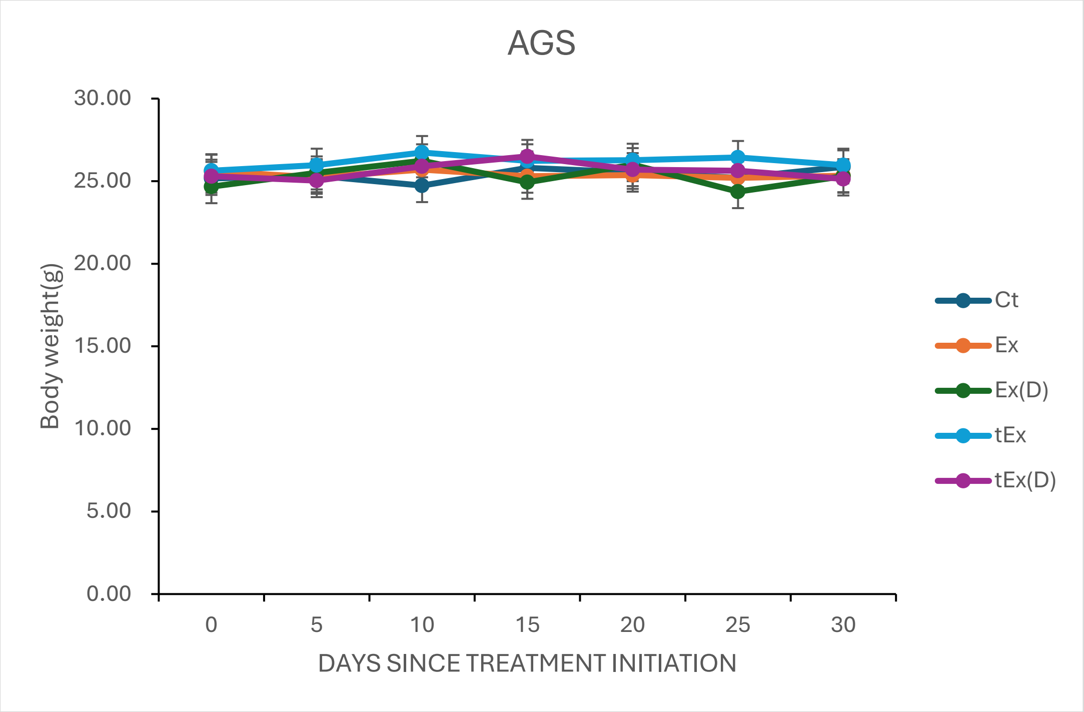
AGS
Body weight(g)
30.00
25.00
20.00
15.00
10.00
5.00
0.00
0
5
10
15
20
25
30
DAYS SINCE TREATMENT INITIATION
Ct
Ex
Ex(D)
tEx
tEx(D)

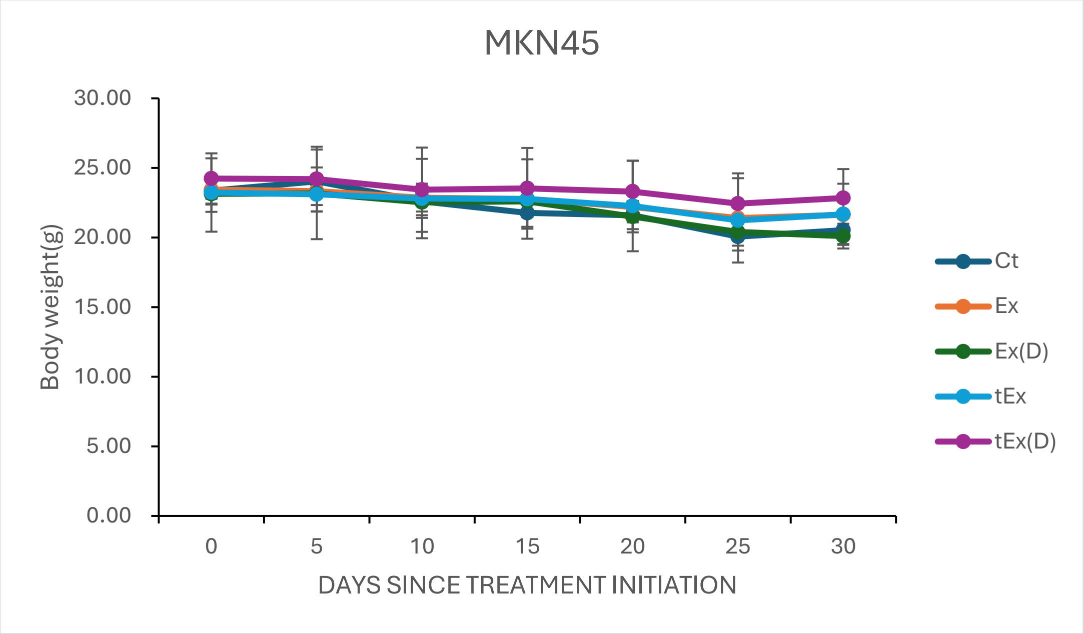
MKN45
Body weight(g)
30.00
25.00
20.00
15.00
10.00
5.00
0.00
0
5
10
15
20
25
30
DAYS SINCE TREATMENT INITIATION
Ct
Ex
Ex(D)
tEx
tEx(D)
